# *Lactobacillus intestinalis* facilitates tumor-derived CCL5 to recruit dendritic cell and suppress colorectal tumorigenesis

**DOI:** 10.1080/19490976.2024.2449111

**Published:** 2025-01-08

**Authors:** Yong Sun, Qiwen Wang, Yao Jiang, Jiamin He, Dingjiacheng Jia, Man Luo, Wentao Shen, Qingyi Wang, Yadong Qi, Yifeng Lin, Ying Zhang, Lan Wang, Liangjing Wang, Shujie Chen, Lina Fan

**Affiliations:** a Department of Gastroenterology, Second Affiliated Hospital of Zhejiang University School of Medicine, Hangzhou, Zhejiang Province, China; b Institution of Gastroenterology, Zhejiang University, Hangzhou, Zhejiang Province, China; c Department of Gastroenterology, Sir Run Run Shaw Hospital, Zhejiang University School of Medicine, Hangzhou, Zhejiang Province, China; d Department of Nutrition, Sir Run Run Shaw Hospital, Zhejiang University School of Medicine, Hangzhou, Zhejiang Province, China; e Prevention and Treatment Research Center of Senescent Disease, Zhejiang University School of Medicine, Hangzhou, Zhejiang Province, China

**Keywords:** *Lactobacillus intestinalis*, colorectal cancer, dendritic cells, CCL5, NF-κB

## Abstract

Gut microbes play a crucial role in regulating the tumor microenvironment (TME) of colorectal cancer (CRC). Nevertheless, the deep mechanism between the microbiota-TME interaction has not been well explored. In this study, we for the first time discovered that *Lactobacillus intestinalis* (*L. intestinalis*) effectively suppressed tumor growth both in the AOM/DSS-induced CRC model and the *Apc*^Min/+^ spontaneous adenoma model. Our investigation revealed that *L. intestinalis* increased the infiltration of immune cells, particularly dendritic cells (DC), in the TME. Mechanically, the tumor-derived CCL5 induced by *L. intestinalis* recruited DC chemotaxis through the NOD1/NF-κB signaling pathway. In clinical samples and datasets, we found positive correlation between *L. intestinalis*, CCL5 level, and the DC-related genes. Our study provided a new strategy for microbial intervention for CRC and deepened the understanding of the interaction between tumor cells and the immune microenvironment modulated by gut microbes.

## Introduction

Colorectal cancer (CRC) ranks as the third most prevalent cancer globally and is the second leading cause of cancer-related deaths.^1,2^ The tumor microenvironment (TME), a complex system including immunocytes, fibroblasts, blood vessels, and cytokines, plays an important role in tumor initiation and progression.^3^ Immunosuppressive phenotypes of immune cells contribute to the poor survival associate with CRC.^4–6^ This suppressive TME is responsible for the onset, progression, and metastasis of CRC.

Specific bacterial species or combinations have been reported to modulate the carcinogenic process of CRC.^7^ Some probiotics exert anti-tumor effects by promoting infiltration and activation of immune cells that lead to tumor diminish.^8^ For instance, *Lactobacillus rhamnosus* GG (LGG) has been found to attenuate CRC burden through dendritic cell activation-mediated CD8^+^ T cell induction;^9^ *Akkermansia muciniphila* has been shown to inhibited CRC tumorigenesis by inducing CD8^+^ T cell proliferation and activation.^10^ In certain instances, tumor cells themselves can actively participate in the interplay between microbial signals and the alteration of TME.^11^ Consequently, the targeting of the gut microbiome on the tumor immune microenvironment holds potential implications for the prevention and therapeutic strategies in CRC.

The bacterial strain *Lactobacillus intestinalis* (*L. intestinalis*) was originally isolated from rat intestines.^12^ An integrated-omics study revealed a positive correlation between the abundance of *L. intestinalis* and natural killer cell activity,^13^ indicating the potential role of *L. intestinalis* in immunoregulation as a probiotic. In our previous study, *L. intestinalis* was identified as an anti-inflammation strain in colitis through Th17 regulation.^14^ However, the probable involvement of *L. intestinalis* in CRC tumorigenesis remains undiscovered.

In this study, we have elucidated that supplementation with *L. intestinalis* effectively reduced CRC burden in both *Apc*^Min/+^ spontaneous and AOM/DSS-induced CRC murine models. Flow cytometric analysis revealed that *L. intestinalis* administration significantly enhanced immune cell infiltration, particularly dendritic cells (DC). Mechanistically, *L. intestinalis* initiated tumor cells to secrete CCL5, a chemotactic factor for DC, through activation of the NOD1/NF-κB signaling pathway. Our findings shed light on the cancer-protective attributes of *L. intestinalis* against CRC, thus presenting promising prospects for microbial-based therapeutic interventions and deepening our understanding of the intricate interplay between gut microbiota, tumor cells, and the TME.

## Material and methods

### Bacterial strains and culture

Bacterial strain *Lactobacillus intestinalis* was purchased from the American Type Culture Collection (#49335, ATCC, USA) and cultured in De Man, Rogosa and Sharpe (MRS) Medium (HB0384–5, hopebio, China) under an atmosphere of 10% H_2_, 10% CO_2_ and 80% N_2_ in 37°C anaerobic workstations (AW500SG, ELECTROTEK, England). For negative control, commensal bacterial strain *Escherichia coli* MG1655 (ATCC 700926) was cultured in Luria-Bertani (LB) medium (A507002 Sangon Biotech, China) overnight at 37°C.

### Cell culture

The human colorectal cancer cell line RKO was purchased from the American Type Culture Collection (ATCC, USA), and the murine colorectal cancer cell line MC38 was obtained from the National Science and Technology Infrastructure, the National Biomedical Cell-Line Resource (NSTI-BMCR, China). RKO and MC38 cells were cultured in Dulbecco’s Modified Eagle Medium (DMEM, Gibco, China) supplemented with 10% fetal bovine serum (FBS, Gibco, China) and 1% penicillin/streptomycin (#BL505A, Biosharp, China) at 37°C in a humidified 5% CO_2_ atmosphere.

### Co-culture of cell and bacteria

For the co-culture assays, MC38 cells were seeded in 6-well plates at a density of 5 × 10^5^ cells per well in DMEM supplemented with 10% FBS and cultured overnight. Subsequently, *L. intestinalis* at a multiplicity of infection (MOI) = 100:1 was added to the plate for co-culture over the following 24 hours. All procedures were conducted at 37°C in a humidified 5% CO_2_ atmosphere.

### Animals use and care

C57BL/6 mice were obtained from Shanghai SLAC Laboratory Animal, China, and *Apc*^Min/+^ mice were purchased from Nanjing Biomedical Research Institute of Nanjing University (NBRI), China. *Itgax*-DTR mice (#NM-KI-204992) were purchased from Shanghai Model Organisms Center, Inc. Animals were housed in Zhejiang Chinese Medical University (ZCMU) Laboratory Animal Research Center in specific pathogen-free (SPF) conditions, with a 12-h light/dark cycle. All the animal experiments were approved by the Institutional Animal Care and Use Committee of ZCMU (IACUC-20220221-12).

### Mouse CRC models

Before administration of bacteria strains, mice were provided with drinking water *ad libitum* for 7 days containing antibiotics (ABX) of 0.2 mg/mL ampicillin (#MB1507, Meilunbio), neomycin sulfate (#MB1716, Meilunbio), metronidazole (#MB2200–1, Meilunbio), and 0.1 mg/mL vancomycin (#MB1260, Meilunbio) to homogenize the gut microbiome and facilitate the colonization of *L. intestinalis*. Subsequently, mice acquired daily intragastric administration with 200 μL PBS, *E. coli* MG1655, or *L. intestinalis* (10^9^ CFU per mouse suspended in 200 μL PBS) as needed.

To employ the AOM/DSS-induced CRC model, 8-week-old C57BL/6 mice were used as previously described.^15^ In brief, following intraperitoneal injection of 10 mg/kg azoxymethane (AOM, #A5486, Sigma) dissolved in normal saline, three cycles of 2.5% dextran sulfate sodium salt (DSS, #0216011080, MP Biomedicals) in the drinking water for 7 days was initiated, separated by a 14-day interval between each cycle. Mice were orally administrated daily with PBS, *E. coli* MG1655, or *L. intestinalis* in the all period of the animal model.

*Apc*^Min/+^ spontaneous adenoma model was utilized to mimic human spontaneous colorectal cancer.^16^ The 8-week-old *Apc*^Min/+^ mice were randomly divided into two groups and administrated with PBS or *L. intestinalis* after antibiotics treatment and sacrificed at day 90.

### siRNA and transfection

MC38 cells were cultured to 60% density, after which the medium was replaced with FBS-free DMEM. For each well of 24-well plate, 40 pmol of siRNA dissolved in RNase-free water and 2 μL of GP-transfect-mate (#G04009, GenePharma) were respectively added to 50 μL of FBS-free DMEM and incubated at room temperature for 5 minutes. Subsequently, the GP-transfect-mate-DMEM mixture was added dropwise to the siRNA-DMEM mixture and incubated at room temperature for 15 minutes. Thereafter, 100 μL of the above mixture was added into each well for a 6-hour incubation period before changing the medium to DMEM supplemented with 10% FBS. The sequence of siRNA was shown in Table S1.

### Subcutaneous tumor model

The subcutaneous tumor model was established as reported.^17^ In brief, MC38 cells were collected and resuspended to 2 × 10^7^ cells/mL in Matrigel matrix (#354234, Corning Biocoat), and subcutaneously injected into C57BL/6 mice. The volume of tumors was measured with the following formula: *V * = 0.5 × *L* × *W* ^2^ (*L*, the longest diameter; *W*, the shortest diameter). In some experiments, MC38 cells were treated with PBS or *L. intestinalis* for 24 hours before harvest for injection.

In the DC-deficient model, *Itgax*-DTR mice received intraperitoneal injection of 4 mg/kg diphtheria toxin (#D381867, Aladdin Biomedical) to deplete DC expressing *Itgax* (CD11c).^18,19^ We then subcutaneously injected MC38 cells co-cultured with or without *L. intestinalis* for 24 hours into the mice, followed by administration of diphtheria toxin every three days until sacrifice.

In the *Ccl5*-knockdown model, MC38 cells were first *in vitro* transfected with si*Ccl5* or non-target siRNA as negative control. Starting from 6 days post injection, mice were intratumorally injected with the complex containing 17.5 μg si*Ccl5* or negative control with 5 μL *in vivo* transfect mate (#G04026, GenePharma) every 3 days until sacrifice.

### Histopathological analysis

The colon of the mouse with the tumor was fixed in paraformaldehyde overnight, embedded in paraffin, and sliced into 5um-thick sections perpendicular to the intestinal tract for subsequent histological staining. Hematoxylin-Eosin (H&E) staining assay was performed with H&E Stain Kit (#G1120, Solarbio, China).

For immunohistochemistry, the paraffin-embedded sections were deparaffinized, antigenically repaired, and incubated overnight at 4°C with anti-Ki67 antibody (1:200, #9129, Cell Signal Technology, USA), anti-CD31 antibody (1:2000, #ab182981, Abcam, China), or anti-CD11c antibody (1:300, #97585, Cell Signal Technology, USA) followed by goat anti-rabbit rabbit (IgG) secondary antibody-HRP (1:2000, #ab205718, Abcam, China) incubation at 37°C for 45 minutes. The stained sections were visualized by DAB staining (#DAB-0031, MXB Biotechnologies, China) according to the manufacturer’s instructions. Histochemistry scoring was calculated according to the formula: H-score = (% strong intensity area × 3) + (% moderate intensity area × 2) + (% weak intensity area × 1).

### Immunofluorescence cell staining

Cells after 24-hour co-culture with PBS or *L. intestinalis* were fixed with 4% paraformaldehyde at room temperature, permeabilized with 0.5% Triton X-100, and stained with anti-CCL5 antibody (1:100, #AF478, R&D systems, USA) overnight at 4°C. DyLight 488 conjugated rabbit anti-goat antibody (1:200, #FDO161, Fdbio science, China) was applied for detection and 4′,6-diamidino-2-phenylindole (DAPI, #P0131, Beyotime, China) for nuclear stain. Images were captured using a fluorescence microscope with a digital camera (#DM4000, Leica, Germany).

### Bone marrow derived dendritic cells (BMDC)

BMDC extraction was conducted following previously reported methods.^20,21^ Briefly, 8-week-old mice were sacrificed and sterilized in 75% ethanol for 15 minutes. Tibiae and fibulae were then isolated, and the bone marrow cavities were washed using RPMI-1640 medium (Gibco, China) supplemented with 10% FBS (Gibco) and 1% penicillin/streptomycin (#BL505A, Biosharp, China). Cells were collected, centrifuged, and resuspended in a complete medium supplemented with 20 ng/ml GM-CSF (#CK02, Novoprotein, China) and 10 ng/ml IL-4 (#CK15, Novoprotein, China). The medium was 50% changed every two days until day 6, after which cells were digested and resuspended once for further maturation.

### DC migration assays

Transwell assays were conducted based on a previous report^22^ to assess the migration capacity of BMDC. The isolated BMDC were seeded in the upper wells of Transwell plates (#3422, Corning, USA). The lower chambers were then filled with 1) 500 μL fresh medium with PBS, *E. coli* MG1655, or *L. intestinalis* (1 × 10^7^ CFU suspended in 25 μL PBS), 2) 1 × 10^5^ MC38 cells suspended in 500 μL fresh medium with PBS, *E. coli* MG1655, or *L. intestinalis* (1 × 10^7^ CFU suspended in 25 μL PBS), or 3) co-culture supernatant of 1 × 10^5^ MC38 cells with PBS, *E. coli* MG1655, or *L. intestinalis* (1 × 10^7^ CFU suspended in 25 μL PBS). After 24 hours, the cells on the lower surface of the upper chamber were fixed overnight with 4% paraformaldehyde (#P0099, Beyotime, China), and afterward were stained with crystal violet (#C0121, Beyotime, China) and photographed using a white-light microscope for further analysis.

### Flow cytometry analysis

The isolation of colorectal lamina propria cells was performed as previously described.^23^ Briefly, the posterior 1/3 of the colorectum without lymphatic and adipose tissue was split into small pieces and incubated in D-Hanks (#MA0039, Meilunbio) buffer, supplemented with 1 mmol/L DTT (#MB30471, Meilunbio) and 5 mmol/L EDTA (#C0196, Beyotime) on a shaker (200 rpm) at 37°C for 30 minutes. Subsequently, the remaining tissue was cut into 1 mm fragments and further digested for 30 minutes in Hanks buffer (#MA0041, Meilunbio) supplemented with 1 mg/mL type IV collagenase (#A005318, Sangon Biotech) on the shaker for 30 minutes. After complete digestion, pass the cell suspension through a 300-mesh filter and centrifuge it at 500 g for 5 minutes.

Isolated cells were counted as 5 × 10^6^ cells/100 μL for each sample, then incubated with purified CD16/32 antibody (101320, Biolegend) for Fc receptors blockade, and with Fixable Viability Stain 510 (FVS510, Cat. #564406, BD Biosciences) for live cell staining. After termination of live staining, the surface molecules of cells were stained in 50 μL Flow Cytometry Staining Buffer for 45 min at 4°C with the following fluorescein-labeled antibodies: Alexa Fluor 700 anti-mouse CD45 (#103128, BioLegend), FITC anti-mouse CD3e (#553061, BD Biosciences), BV711 anti-mouse NK-1.1 (#740663, BD Biosciences), BV605 anti-mouse CD4 (#100548, BioLegend), APC-Cy7 anti-mouse CD8a (#561967, BD Biosciences), PE/Dazzle™ 594 anti-mouse Ly-6 G (#127648, BioLegend), BUV395 anti-CD11b (#563553, BD Biosciences), BV421 anti-mouse F4/80 (#123132, BioLegend), PE anti-mouse I-A/I-E (#557000, BD Biosciences), PE/Cyanine7 anti-mouse CD11c (#117318, BioLegend), BV785 anti-mouse CD69 (#104543, BioLegend), BB700 anti-mouse CD279 (PD-1) (#566514, BD Biosciences).

### Isolation process of *L.* *intestinalis* crude extraction

The extraction of crude components of *L. intestinalis* was performed according to previous studies.^24,25^ In brief, *L. intestinalis* was cultured in MRS medium until reaching the exponential growth phase. After centrifugation, the supernatant of culture medium was collected and the pH was adjusted to 7.0 to obtain the conditional medium of *L. intestinalis* (*L. int*-CM). Subsequently, the pellet was subjected to ultrasonic lysis and centrifugation, and the resulting supernatant and precipitation were then collected for co-culture assays.

### RNA extraction and quantitative real-time PCR

RNA from mouse colorectal tumors or cells were extracted with Trizol reagent (Invitrogen, USA) and RNA extraction kit (#AG21024, Accurate Biology, China). ABScript III RT Master Mix (#RK20429, ABclonal, China) was used for reverse transcription. RT-qPCR reaction mix contained Universal SYBR Green Fast qPCR Mix (#RK21203, ABclonal, China), specific primers (Table S2), and cDNA. RT-qPCR was performed under the following procedure: 95°C for 2 min, followed by 50 cycles of 95°C for 15 sec and 60°C for 30 sec, in Light Cycler®480 Real-Time PCR System (Roche). The relative mRNA expression level was analyzed using the -ΔCt method or the 2^−ΔΔCt^ method. The mouse *Actb* gene or human *ACTB* gene was used as an internal reference.

### DNA extraction and bacteria quantification

Bacterial DNA from mice or human feces was extracted using TIANGEN fecal genome extraction kits (#DP328–02, TIANGEN, China), and bacterial DNA from human tissues or mice tumor tissues was extracted using TIANGEN genome extraction kits (#DP304–02, TIANGEN, China). RT-qPCR reaction mix consists of Universal SYBR Green Fast qPCR Mix (#RK21203, ABclonal, China), primers (Table S2), and template DNA. RT-qPCR assays were performed in ROCHE LightCycler®480 System (Rotor gene 6000 Software, Sydney, Australia). Relative abundance was calculated with the -ΔCt method and *universal Eubacteria* 16S was detected as an internal reference gene.^26^

### RNA sequencing

MC38 cells were co-cultured with PBS or *L. intestinalis* (MOI 100:1) for 24 hours. Thereafter, total RNA was extracted from cells by Trizol reagent (Invitrogen, USA). Library construction and sequencing were carried out at Majorbio Bio-pharm Biotechnology Co., Ltd. (Shanghai, China) following the manufacturer’s guidelines. Expression levels were determined by FPKM (fragments per kilobase per million) and DESeq2 was used to perform the differential expression analysis. A significance level of *p* < 0.05 and |log2 (Fold Change)| > 1 were deemed significant. The “pheatmap” and “ggplot2” R packages were utilized to create the heatmap plot and MA plot, and KEGG enrichment analysis was conducted using the “clusterProfiler” R package.

### Enzyme-linked immunosorbent assay (ELISA)

C–C motif chemokine ligand 5 (CCL5) proteins in the cell culture media were quantified using cytokine-specific ELISA kits (#abs520014, Absin Bioscience, China) in accordance with the manufacturer’s guidance. To elaborate, antibodies were precoated onto 96-well plates, and either 100 μL of the test supernatant or a certain dilution of the standard was added and incubated at room temperature for 2 hours. Following the washing procedure, the biotinylated secondary antibody was added to the wells and incubated for two hours. After three additional washes, streptavidin solution and the chromogenic substrate were added, then the intensity at 450 nm and 540 nm was measured. The sample concentration was determined using the standard curve of OD450-OD540 and concentration.

### Western blot analysis

Proteins from MC38 cells were extracted with Cell lysis buffer for Western and IP (#P0013, Beyotime, China), and quantified using a BCA protein assay kit (#FD2001, Fdbio science, China). Proteins were separated in 10%-12% sodium dodecyl sulfate-polyacrylamide (SDS-PAGE) gels and then transferred to PVDF membranes. Then, membranes were blocked with Quickblock solution (P0231, Beyotime, China) for 20 minutes, and incubated with the following primary antibodies overnight at 4°C: CCL5 antibody (1:1000, #AF478, R&D systems, USA), NF-κB p65 (1:1000, #8242, Cell Signaling Technology, USA), Phospho-NF-κB p65 (1:1000, #3033, Cell Signaling Technology, USA), β-Actin (#81115–1-RR, Proteintech, China). Subsequently, the membranes were incubated with secondary antibody-conjugated HRP (1:5000, HuaBio, China) for 2 hours at room temperature, and bands were visualized using an ECL kit (#P10300, NCM Biotech, China).

### Human sample collection

All human samples including feces and tissues were obtained from Sir Run Run Shaw Hospital, Zhejiang University School of Medicine, which has been approved by the Clinical Research Ethics Committee of Sir Run Run Shaw Hospital, Zhejiang University School of Medicine (Approval NO. 2022–0430).

The stool samples and their pathological information were obtained from 104 patients with colorectal cancer (CRC), 85 patients with colorectal adenoma (CRA), and 77 healthy participants with no history of CRC or CRA. Tissue samples of colorectal cancer were obtained from 36 patients by colonoscopy or surgery, and confirmed with pathological examination. All samples were collected carefully and immediately frozen in liquid nitrogen.

### Immune score analysis

RNA-sequencing expression profiles of patients with colorectal cancer were downloaded from the TCGA dataset (https://portal.gdc.com). Samples were divided into *CCL5* high group and *CCL5* low group according to the expression of *CCL5*. R package “immuneeconv” was utilized to assess the immune infiltration by algorithms of “xCell” which had been benchmarked.^27,28^

### Statistical analysis

Statistical analysis was performed using the GraphPad Prism 9.0 and R 4.2.2 software. Unpaired Student’s t test, Mann–Whitney test, one-way ANOVA, Wilcox test, or Spearman correlation analysis were used for statistical analysis as shown in figure legends. *p* value less than 0.05 was considered statistically significant.

## Results

### Oral supplement with *L.* *intestinalis* suppresses colorectal tumorigenesis in mice models

**Figure 1.**
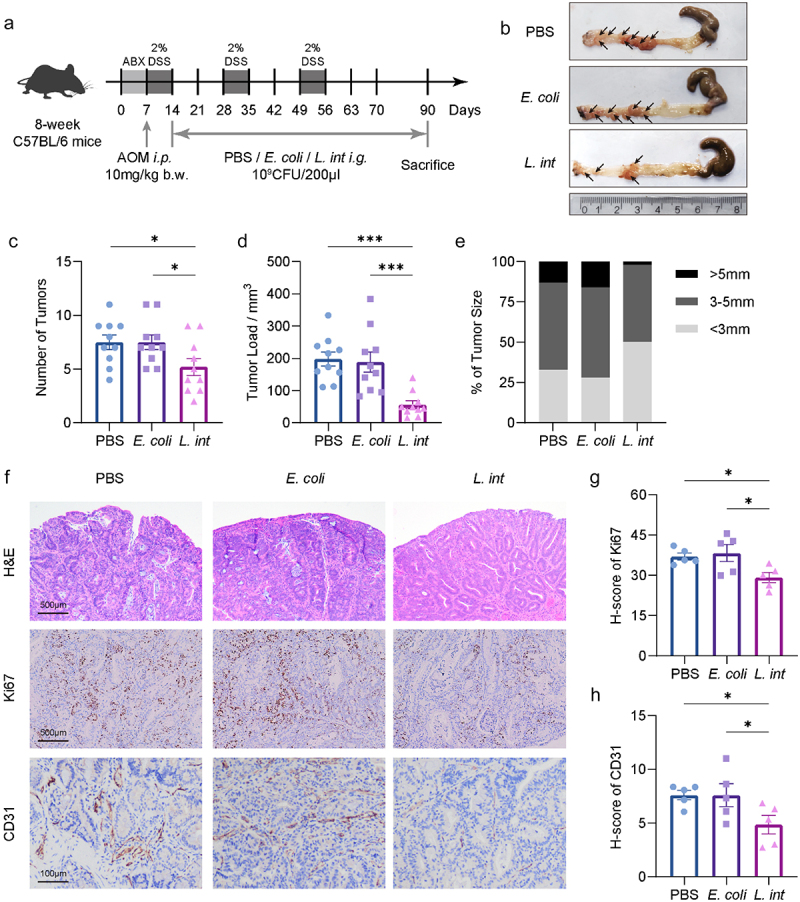
Oral supplement with *L. intestinalis* suppressed colorectal tumorigenesis in AOM/DSS-induced CRC model.

To assess the potential role of *L. intestinalis* in the progression of colorectal cancer (CRC), we employed an azoxymethane (AOM) and dextran sulfate sodium salt (DSS)-induced mice CRC model. Prior to inducing CRC, mice were treated with antibiotics for 7 days followed by oral gavage of *L. intestinalis* (*L. int*), *Escherichia coli* (*E. coli*) or PBS (Figure 1a). We first confirmed the enhanced colonization by quantifying *L. intestinalis* abundance in colon tissue (Figure S1a). Compared to the *E. coli* and PBS control group, we found *L. intestinalis* reduced the number of tumors and alleviated tumor load significantly (Figure 1b–e). Furthermore, we observed that *L. intestinalis* administration led to a reduction in the neoplasms within colorectal tumors by H&E staining, and decreased expression levels of the proliferation marker Ki67 and blood vessel invasion marker CD31 by immunohistochemistry (Figure 1f–h).

(a) Schematic diagram of AOM/DSS-induced CRC model supplemented with *L. intestinalis*, *E. coli*, or control. (b–e) Representative tumor-bearing colon images (b), numbers of tumors per colon (c), tumor loads (d), and tumor size (e) of the groups supplemented with PBS, *E. coli* MG1655, or *L. intestinalis* were shown. *n* = 9-10 per group. (f) The tumor dysplasia examined by H&E staining, the proliferation marker Ki67 immunohistochemistry, and the blood vessel invasion marker CD31 immunohistochemistry in the tumor tissue of AOM/DSS-induced CRC model treated by PBS, *E. coli* MG1655 or *L. intestinalis*. Scale bars, 500 or 100 μm. (g-h) The quantitative scores of Ki67 (g) and CD31(h), *n* = 5 per group. Data are shown as mean ± SEM; *p < 0.05; ***p < 0.001; ANOVA test (c-d, g-h). AOM, azoxymethane; DSS, dextran sodium sulfate; i.p., intraperitoneal injections; b.w., body weight; PBS, phosphate buffered saline; H&E, hematoxylin and eosin; *L. int*, *L. intestinalis*; *E. coli, Escherichia coli.*

Additionally, we conducted a spontaneous adenoma model using *Apc*^Min/+^ mice to further evaluate the function of *L. intestinalis* in colorectal tumors (Figure S1b). Consistently, intragastric gavage with *L. intestinalis* resulted in a reduction in both the number and size of colon neoplasms (Figure S1c). Taken together, these data collectively indicated that supplementation with *L. intestinalis* suppressed colorectal tumorigenesis *in vivo*.

### *L.* *intestinalis* recruited dendritic cells in AOM/DSS-induced CRC model

To investigate the underlying mechanisms by which *L. intestinalis* inhibited tumorigenesis, we performed *in vitro* cell proliferation assays to evaluate the suppressive effects on tumor cell lines. The results revealed that *L. intestinalis* did not exhibit an inhibitory effect on cell proliferation in human CRC cell line RKO or mouse CRC cell line MC38 (Figure S2a,b). We then utilized the supernatant of bacterial culture (conditioned medium, *L.int*-CM) for co-culturing with MC38 cells to further investigate the influence of bacterial metabolites on CRC cell proliferation. However, the addition of 2.5% or 5% *L.int*-CM did not impact the proliferation of tumor cells either (Figure S2c,d).

**Figure 2.**
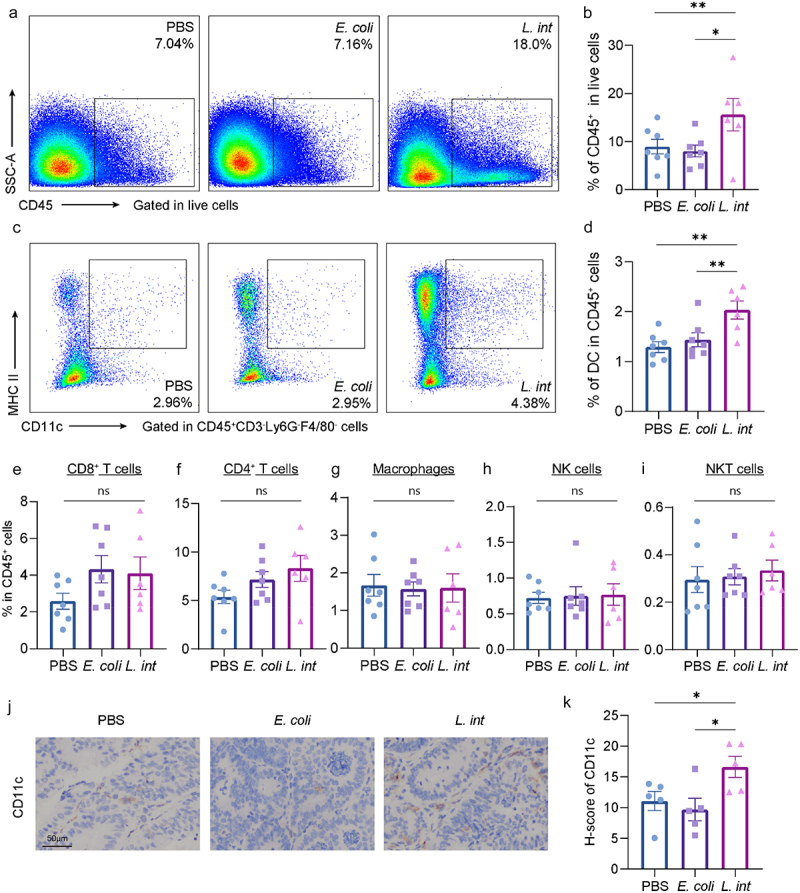
*L. intestinalis* recruited dendritic cells into the tumor microenvironment in the AOM/DSS-induced CRC model.

Consequently, we shifted our focus on the tumor microenvironment (TME) and explored the immune response in the colon tissues from the AOM/DSS model by flow cytometry analysis (Figure S3a). Remarkably, we found that the number of all immune cells was noticeably higher in the *L. intestinalis* group compared to the other two groups (Figure 2a,b), suggesting that *L. intestinalis* induced a significant immune infiltration. Among the major clusters of immune cells, the most notable increase was observed in DC (Figure 2c–i). Furthermore, we further found the increased protein level of CD11c (a DC marker) in tumor tissue after *L. intestinalis* treatment via immunohistochemistry (Figure 2j–k) and observed the upregulated mRNA expression level of DC-related genes *Itgax*, *Flt3*, and *Batf* in the *L. intestinalis* group in the tumor tissues of the AOM/DSS model (Figure S4a). Additionally, we observed similar changes in the flow cytometry results of the *Apc*^Min/+^ model (Figure S4b).

(a–b) Percentage of total immune cells (CD45^+^ cells) in colonic lamina propria of AOM/DSS-induced CRC model tested by flow cytometry. (c–d) Percentage of dendritic cells (CD11c^+^MHCII^+^ cells) in total immune cells of colonic lamina propria. (e–i) Percentage of CD8^+^ T cells (e), CD4^+^ T cells (f), macrophages (g), natural killer cells (h), and natural killer T cells (i) in total immune cells of colonic lamina propria, *n* = 6-7 per group. (j) The protein level of CD11c in the tumor tissue from AOM/DSS-induced CRC was examined by immunohistochemistry staining. Scale bar, 50 μm. (k)The quantitative score of CD11c immunohistochemistry, *n* = 5 per group. Data are presented as mean ± SEM; *p < 0.05; **p < 0.01; ns, no significance. ANOVA test (b, d-i, k). *L. int*, *L. intestinalis.*

Despite that there was no significant change in CD8^+^ T cells (Figure 2e), we surprisingly found that the percentage of CD69^+^CD8^+^ T cells (activation marker of CD8^+^ T cells) was increased in the *L. intestinalis* group (Figure S4c,d), and it was positively related to the percentage of DC in immune cells (Figure S4e). Oppositely, the expression of inhibitory marker PD-1 was decreased in CD8^+^ T cells in the *L. intestinalis* group (Figure S4f,g). These above results showed that *L. intestinalis* remodeled an anti-tumor immune response by increasing the infiltration of DC and activating CD8^+^ T cells.

### *L.* *intestinalis* enhanced tumor cells to secret CCL5 to promote DC chemotaxis and suppress tumorigenesis

**Figure 3.**
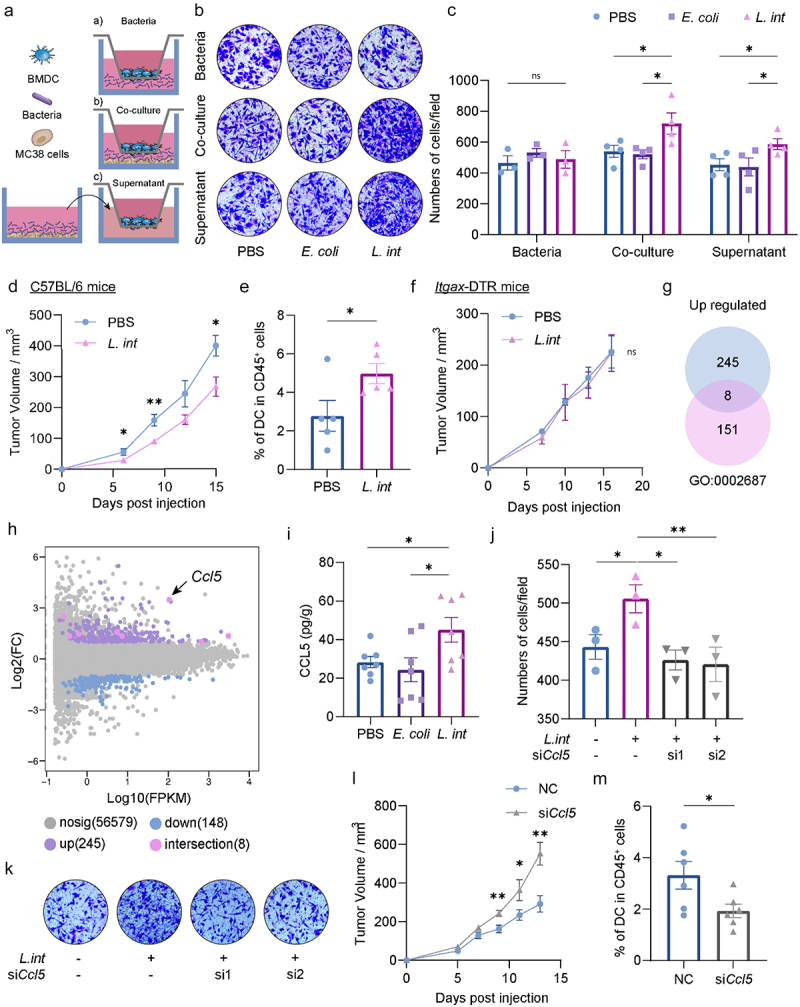
*L. intestinalis* induced colorectal tumor cells to secret CCL5 to promote DC chemotaxis and suppress colorectal tumorigenesis.

Tumor-infiltrating DC originate from myeloid progenitor cells in the bone marrow and are transported to the tumor microenvironment.^29^ Therefore, we conducted Transwell assays to investigate the impact of *L. intestinalis* on the migration capacity of DC (Figure 3a). We observed that *L. intestinalis* itself did not promote DC migration; however, DC migration was observed when *L. intestinalis*-treated MC38 cells were placed in the lower chamber. Moreover, similar results were obtained when adding the supernatant from MC38 cells co-cultured with *L. intestinalis* (Figure 3b,c). Subsequently, we subcutaneously implanted MC38 cells co-cultured with or without *L. intestinalis* on C57BL/6 mice. The *L. intestinalis* group exhibited both inhibited tumor growth and increased DC infiltration (Figure 3d,e). These findings might suggest that chemoattractant secreted by tumor cells mediated the migration of DC and ultimately led to inhibition of tumor growth.

(a–c) Transwell assays of BMDC co-cultured with bacteria or tumor cells. BMDC were plated on the upper chamber and then co-cultured by three types of conditions in the underlayer: a) Bacteria alone: PBS, *E. coli* MG1655 or *L. intestinalis* (*L. int*) in the underlayer; b) Co-culture with bacteria and cancer cells: MC38 cells with PBS, *E. coli* MG1655 or *L. intestinalis* (*L. int*) in the under layer; c) supernatant treatment: MC38 cells were treated by PBS, *E. coli* MG1655 or *L. intestinalis* (*L. int*), then the supernatants were applied to the BMDC cells. Schematic diagram (a), representative Transwell images (b), and statistical analysis (c) of migrating cells were shown, *n* = 3-4 per group. (d–e) MC38 cells were co-cultured with PBS or *L. intestinalis* for 24 hours, then injected into the right leg of C57BL/6 mice (*n* = 5 per group). Tumor volume was recorded (d), the infiltrated dendritic cells in the tumor tissue were analyzed by flow cytometry (e). (f) MC38 cells co-cultured with PBS or *L. intestinalis* were injected into *Itgax*-DTR mice, and the tumor volume was measured. (g) The intersection of upregulated genes in MC38 cells treated with *L. intestinalis* and the ‘Positive Regulation of Leukocyte Migration’ Gene Set (GO:0002687) were shown by the Venn diagram. (h) The differential gene expression and the intersection genes in Figure 3g were shown by MA plot (*n* = 3 per group, fold change > 2, FPKM > 1, FDR < 0.05). (i) The concentration of CCL5 in the tumor of AOM/DSS-induced CRC murine model was measured by ELISA (*n* = 7). (j–k) Statistic analysis (j) and representative Transwell images (k) of BMDC, when co-cultured with MC38 cells treated by PBS or *L. intestinalis* with or without si*Ccl5* treatment in the under layers (*n* = 3). (l) Tumor volume curve of MC38 cell-derived xenograft transfected with or without si*Ccl5* (*n* = 6). (m) The infiltrated dendritic cells in the tumor tissue of *in vivo* si*Ccl5* model were analyzed by flow cytometry (*n* = 6). Data are presented as mean ± SEM; *p < 0.05; **p < 0.01; ns, no significance. ANOVA test (c, i-j), Student’s t test (d-f, l-m). BMDC, bone marrow-derived dendritic cells; NC, negative control.

Moreover, we used *Itgax*-DTR mice to establish a dendritic cell (DC)-deficient mouse model,^18,19^ in which diphtheria toxin receptor (DTR) is co-expressed with *Itgax* gene. DCs expressing *Itgax* (encoding CD11c) were depleted by intraperitoneal injection of diphtheria toxin throughout the experiment, as confirmed via flow cytometry (Figure S5a). We then subcutaneously injected MC38 cells treated with or without *L. intestinalis* into the *Itgax*-DTR mice (Figure S5b). The tumor growth inhibition induced by *L. intestinalis* was diminished in the *Itgax*-DTR mice (Figure 3f). These findings revealed the critical role of DC in mediating the tumor-suppressive effects of *L. intestinalis*.

To determine the chemoattractant that recruited DC, we further performed RNA sequencing on MC38 cells co-cultured with *L. intestinalis* or PBS control and observed 253 up-regulated genes and 148 down-regulated genes in the *L. intestinalis* group (Log (2) Fold change > 2, *p* < 0.05), and then we defined 8 mainly concerned genes (Figure 3g) by intersecting the up-regulated genes with the ‘positive regulation of leukocyte migration’ gene set (GO:0002687). Among the 8 intersected genes, *Ccl5* had a prominent contribution as the MA plot showed (Figure 3h). We further demonstrated that supplement of *L. intestinalis* increased the expression level of CCL5 in the tumor tissue of AOM/DSS-induced CRC model through ELISA (Figure 3i) and RT-qPCR (Figure S5c). Furthermore, we confirmed that *L. intestinalis* increased the expression of CCL5 *in vitro* by RT-qPCR, western blotting, and immunocytochemistry (Figure S5d,e). In addition, we observed a similar up-regulation effect on human CRC cell line RKO (Figure S5f).

CCL5 (C–C motif chemokine ligand 5) is a chemokine that exhibits activity on dendritic cell migration.^30^ Several studies have demonstrated that CCL5 secretion in the TME had an effective anti-tumor therapy.^31,32^ We hypothesized that the *L. intestinalis*-induced DC recruitment relied on the upregulation of CCL5 in tumor cells. Thus, we knocked down the expression of the *Ccl5* gene on MC38 cells through siRNA transfection (Figure S5g). MC38 cells transfected with si*Ccl5* displayed a diminished capacity to attract migration of DC, as compared to the negative control group (Figure 3j,k). Subsequently, to further demonstrate the effect of CCL5 *in vivo*, we subcutaneous injected MC38 cells transfected with si*Ccl5* to conduct a subcutaneous tumor model. Thereafter we maintained *Ccl5* knockdown through intratumorally injection of si*Ccl5* every three days (Figure S5h). We measured the concentration of CCL5 in tumor site by ELSA to confirm the knockdown efficiency (Figure S5i). As shown in Figure 3l,m, knockdown of *Ccl5* resulted in augmented tumor growth and diminished infiltration of DC.

Our findings provided evidence that *L. intestinalis* induced tumor cells to secrete CCL5, thereby facilitating the chemotaxis of DC and suppressing the tumorigenesis.

### NOD1/NF-κB pathway participated in *L.* *intestinalis*-activated CCL5 secretion

**Figure 4.**
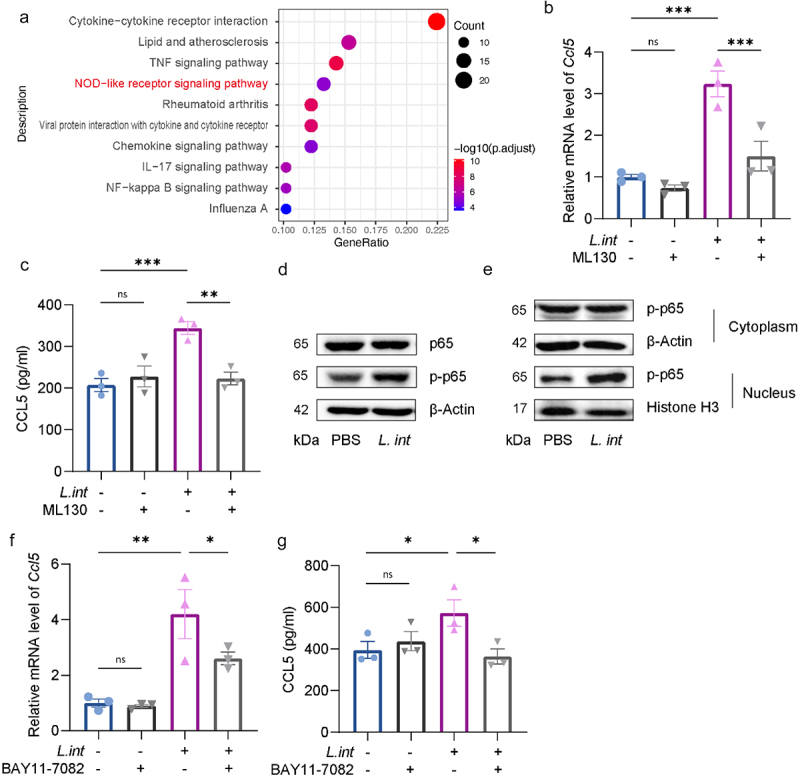
NOD1/NF-κB pathway participated in *L. intestinalis*-activated CCL5 secretion.

To further explore the mechanism involved in *L. intestinalis* activated CCL5 secretion in tumor cells, we performed KEGG enrichment analysis for the 253 up-regulated genes, revealing the top 10 enriched pathways (Figure 4a). There are various pathways involved in the interaction between bacteria and host cells, and at last, we focused on the NOD-like receptor (NLR) signaling pathway for deep investigation. Previous studies reported NOD1 and NOD2 as the receptors that could initiate the NLR pathway.^33^ Therefore, we examined the expression level of the *Nod1* and *Nod2* genes in tumor tissue of the AOM/DSS model by RT-qPCR. The results revealed that gavage of *L. intestinalis* could increase the mRNA expression level of *Nod1* but not *Nod2* (Figure S6a). To further affirm the participation of the NOD1 receptor in CCL5 secretion activated by *L. intestinalis*, we used NOD1-specific inhibitor ML130 to block the pathway. It was observed that ML130 diminished the increased expression of CCL5 activated by *L. intestinalis* via RT-qPCR and ELISA assays (Figure 4b,c).

(a) KEGG enrichment analysis of upregulated genes of MC38 cells after co-culturing with *L. intestinalis*. (b–c) The expression of CCL5 in MC38 cells when co-cultured with *L. intestinalis* or PBS control, with or without 0.5 μM ML130 treatment, respectively quantified by RT-qPCR (b) and ELISA (c) (*n* = 3). (d) The protein expression level of p65 and phospho-p65 was tested by western blotting in MC38 cells with or without *L. intestinalis* treatment. (e) The protein expression level of phospho-p65 in the cytoplasm and nucleus of MC38 cells was tested by western blotting. (f-g) MC38 cells were co-cultured with *L. intestinalis* or PBS control with or without 2.5 μM BAY11-7082 treatment, then the expression of CCL5 was quantified by RT-qPCR (f) and ELISA (g) (*n* = 3). Data are presented as mean ± SEM; *p < 0.05; **p < .01; ***p < .001; ns, no significance. ANOVA test (b-c, f-g).

Previous studies reported some pathways involved in the NOD1 receptor signal conduction, like NF-κB, MAPK pathways etc.^33^ We detected the key proteins and their phosphorylation levels in the NF-κB pathway (p65) or the MAPK pathway (p38 and Jnk) using western blotting, and then we observed an elevated level of phosphorylated p65, as well as an increased translocation into the nucleus from the cytoplasm (Figures 4d,e, S6b,c). In contrast, no obvious change was observed in the phosphorylation levels of p38 and Jnk (Figure S6d). Moreover, we utilized BAY 11–7082, an NF-κB pathway inhibitor, and found that BAY 11–7082 decreased the elevated activation of CCL5 by *L. intestinalis* on both mRNA and protein levels. (Figure 4f,g).

To investigate the crude component activates NOD1/NF-κB pathway and enhances the CCL5-secreting phenotype of tumor cells, we then collected the conditional medium of *L. intestinalis* (*L. int*-CM) and subsequently lysis the bacteria pellet with ultrasonic followed by centrifugation to preliminary separation the bacterial fractions as supernatant and precipitation (Figure S6e). Upon co-culturing these fractions with MC38 cells, we observed that *L. int*-CM and supernatant of ultrasonic lysis did not enhance the expression of CCL5 (Figure S6f,g), while the precipitation exhibited the most pronounced upregulation (Figure S6h,i).

Taken together, these results suggested the NOD1/NF-κB pathway was involved in the secretion of CCL5 from tumor cells activated by *L. intestinalis*.

### The abundance of *L.* *intestinalis* was positively correlated with *CCL5, ITGAX*, and *NOD1* in CRC patients

**Figure 5.**
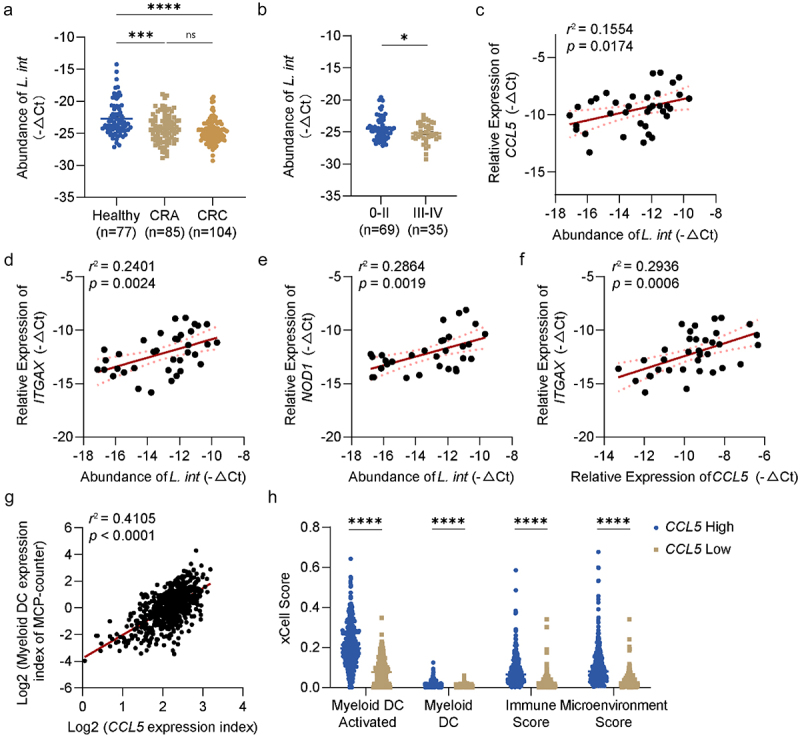
The abundance of *L. intestinalis* was positively correlated with *CCL5*, *ITGAX*, and *NOD1* in CRC patients.

To assess the value of *L. intestinalis* in clinical applications, we collected stool and tumor tissue samples from clinical participants for further investigation. In the first cohort, stool samples from 104 patients diagnosed with CRC, 85 patients diagnosed with colorectal adenoma (CRA), and 77 healthy participants were used for bacterial genome extraction and RT-qPCR analysis. It was observed that the abundance of *L. intestinalis* decreased in both CRA and CRC patients (Figure 5a). Furthermore, we found that patients with advanced diseases had lower levels of *L. intestinalis* (Figure 5b).

(a) The fecal abundance of *L. intestinalis* in healthy control and patients with colorectal adenoma or colorectal cancer measured by RT-qPCR. (b) The fecal abundance of *L. intestinalis* in CRC patients at stage 0-II and stage III-Ⅳ. (c–f) The mRNA level of *CCL5*, *ITGAX, NOD1*, and the abundance of *L. intestinalis* in tumor tissue of CRC patients. The correlations of *L. intestinalis* and *CCL5* (c), *ITGAX* (d), and *NOD1* (e), and the correlation of *CCL5* and *ITGAX* (f) were analyzed. (g) The positive correlation of expression index of myeloid dendritic cells and *CCL5*, calculated by MCP-counter based on RNA sequencing profile from TCGA database. (h) The expression distribution of myeloid dendritic cell activated, myeloid dendritic cell, immune score, and microenvironment score in the CCL5-high group and CCL5-low group, calculated by xCell based on RNA sequencing profile from TCGA database. Data are presented as mean ± SEM; *p < 0.05; ***p < .001; ****p < .0001; ns, no significance. ANOVA test (a), Student’s t test (b), Spearman correlation analysis (c-g), Wilcox test (h).

We then collected tumor tissues from 36 patients diagnosed with CRC for bacterial genome extraction and total RNA extraction. It revealed a positive correlation between the abundance of *L. intestinalis* and *CCL5*, *ITGAX*, and *NOD1* genes (Figure 5c–e). Additionally, the DC-related gene *ITGAX* showed a positive correlation with the expression level of *CCL5* (Figure 5f). Furthermore, we examined RNA sequencing data of CRC patients in the TCGA database using two types of algorithms. A significant positive correlation was observed between the level of the *CCL5* gene and the abundance of DC cells calculated by the MCP-counter algorithm (Figure 5g). Similarly, the higher *CCL5* expression group had elevated levels of myeloid DC, higher immuno-scores, and microenvironmental scores calculated using the xCell algorithm (Figure 5h). These results indicated *L. intestinalis* had a clinical significance during the development of CRC, suggesting the possibility of its application in the treatment of CRC patients.

**Figure 6.**
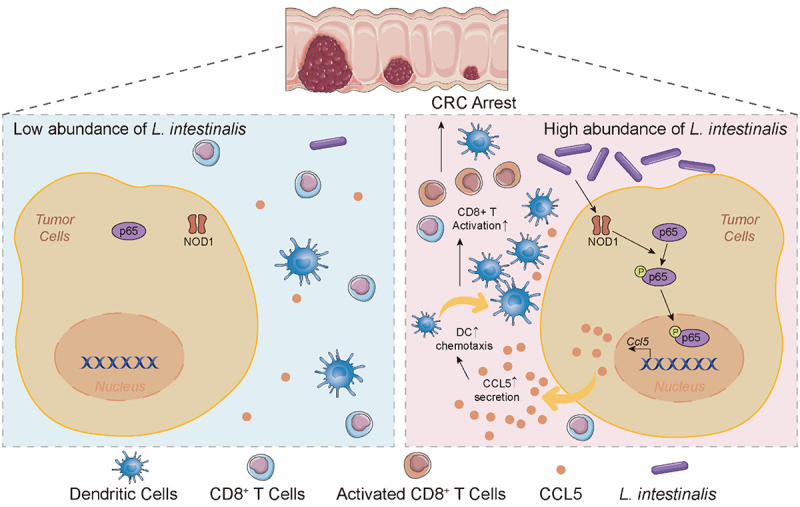
The scheme diagram of *L. intestinalis* suppressing tumorigenesis.

To sum up, in this study, we identify an unreported species *L. intestinalis* as a tumor-suppressing microorganism for CRC. Mechanically, *L. intestinalis* ameliorates tumorigenesis by stimulating tumor cells to secrete CCL5 and recruiting DC in the TME by the NOD1/NF-κB signaling pathway (Figure 6).

The schematic diagram shows the supplementation of *L. intestinalis* inducing tumor-secreted CCL5 and dendritic cell chemotaxis, and suppressing colorectal tumorigenesis.

## Discussion

An increasing number of studies have focused on modulating the TME to mitigate tumor burden by microbial interventions.^34–36^ For instance, our previous studies have demonstrated that *Akkermansia muciniphila* effectively inhibits CRC tumorigenesis by inducing M1-like tumor-associated macrophages,^37^ and *Bifidobacterium adolescentis* was found to activate a cluster of Decorin^+^ macrophages, contributing to the suppression of CRC.^17^ In this study, we have first identified *L. intestinalis* as a tumor-suppressive strain and conclusively demonstrates its crucial functions in the TME.

DC play a supportive role in anti-tumor immunity by antigen-presentation, interacting with cytotoxic T cells to directly eliminate tumor cells, and regulating the tumor immune microenvironment.^38^ Specifically, precursor DC (pre-DC) are recruited from the peripheral circulation and differentiate into conventional DC (cDC) within the TME, collaborating to initiate CD8^+^ and CD4^+^ T cells for an elaborate antitumor effect.^39^ Consequently, strategies aimed to increase DC in the TME have been attempted in clinical cancer trials to improve tumor prognosis, such as through chemotaxis,^40^ DC vaccines,^41^ and proliferation induction.^42^ In our study, we discovered that an augmented quantity of immune cells, especially DC in TME of murine models serves as a pivotal immunomodulatory element contributing to the anti-tumor properties of *L. intestinalis*, and in the clinical cohort, the abundance of *L. intestinalis* is positively correlated with gene marker of DC.

In our investigation, we found that *L. intestinalis* enhanced tumor cell-derived CCL5 secretion to eliminate tumors. However, the impact of CCL5 on tumors is inconsistent in different reports.^43–45^ In the field of CRC research, Courtney et al. underscored the indispensability of CCL5 in anti-tumor immunity as it recruits tumor-infiltrating lymphocytes.^32^ Conversely, Liu et al. discovered that macrophage-derived CCL5 facilitates immune escape by stabilizing PD-L1 on tumor cells.^46^ Given that CCL5 has chemotactic activity for a wide range of immune cells^47,48^ including dendritic cells,^49^ the effect of CCL5 on tumors may depend partially on the diversity of cells producing or responding to CCL5, as well as on immunoreactive substances that it interacts with.

Although the precise mechanisms underlying the CCL5 network need further elucidation, and studies focusing on the inhibitory effects of CCL5 secreted from tumor cells on tumorigenesis remain limited, researchers have been dedicated to investigating CCL5 as a modulator in the tumor microenvironment.^50–53^ This approach has exhibited considerable potential in enhancing immune infiltration and mitigating tumor burdens. We observed that elevating tumor cell-derived CCL5 secretion was responsible for recruiting DC, consequently leading to a reduction in tumor growth. Our findings could support the idea that the increased CCL5 in specific cells serves as an effective and promising adjuvant in tumor treatment.

The NOD1/NF-κB pathway is a common response for microbial signals.^54^ The NOD1 receptor predominantly detects the peptidoglycan fragment γ-D-Glu-mDAP (ie-DAP), which is a component of the microbial cell wall.^55^ As previous studies described, peptidoglycan is predominantly present in the precipitation of ultrasonic lysis.^24,25^ Therefore, we tentatively propose that fragments of the bacterial cell wall component, peptidoglycan, may serve as activators of NOD1 signaling.

The NF-κB signaling pathway is a cellular cascade mechanism whereby upstream signals heighten target gene expression in a p65-dependent way, consequently impacting cell proliferation and inflammatory responses.^56^ Our study demonstrated that the NOD1 receptor is crucial for the *L. intestinalis* signal sensor and consequent molecular events leading to upregulation of CCL5 selection of CRC cells. Besides, we observed that activation of the NOD1/NF-κB signaling pathway by *L. intestinalis* did not have an accelerating effect on tumor cell proliferation.

Multiple recent studies have shed light on the implication of tumor cells in the interaction mechanisms between microbial-tumor and immune microenvironments. For example, bacterial metabolites increase cancer cell expression of HLA class I, resulting in greater responsiveness to immunotherapy.^57^ In another case, microbial signals decrease the production of chemokines produced by tumor cells, reducing the function of CD8^+^ T cells.^11^ Our study emphasizes the significance of the activation of tumor cell signal pathways and the following secretion of chemokines for regulating the tumor immune microenvironment and suppressing tumorigenesis by *L. intestinalis*.

Our study has several limitations. The potential adoption of more refined murine models could offer enhanced validation of our findings, including germ-free mice to ascertain the anti-tumor effect of *L. intestinalis* to delineate the mechanisms, and patient-derived microbiota-transplanted mice to better mimic the clinical status. Our understanding of the TME, particularly regarding the origin and response of CCL5, remains rudimentary, necessitating more comprehensive analyses such as single-cell transcriptomics to provide further insights. Further research is needed to identify the specific components of *L. intestinalis* in activating the NOD1/NF-κB pathway, as well as its uniqueness and combining mechanism with receptors. Notably, isolation of patient-derived *L. intestinalis* strains rather than standardized strains and identifying their functions, as well as establishing comprehensive CRC clinical database hold promise for future clinical applications.

In conclusion, our study confirms the antitumor effects of a new probiotic *L. intestinalis*, which opens extra possibilities for enterobacterial therapy in CRC management. Furthermore, we emphasize the importance of tumor cells that respond to microbial signals in the tumor immune microenvironment, which provides broader insights into understanding the microbe-tumor-TME interaction.

## Supplementary Material

Supplemental information 1109.docx

## Data Availability

The authors confirm that the data supporting the findings of this study are available within the article and its supplementary materials. Source data and reagents are available from the corresponding author on reasonable request.
