# Supplementary material for: Lactobacillus intestinalis facilitates tumor-derived CCL5 to recruit dendritic cell and suppress colorectal tumorigenesis
Source: Gut Microbes. 2025 Jan 8;17(1):2449111. doi: 10.1080/19490976.2024.2449111 (PMC11730368; doi:10.1080/19490976.2024.2449111)
Supplement: Supplemental information 1109.docx [file KGMI_A_2449111_SM3614.docx]

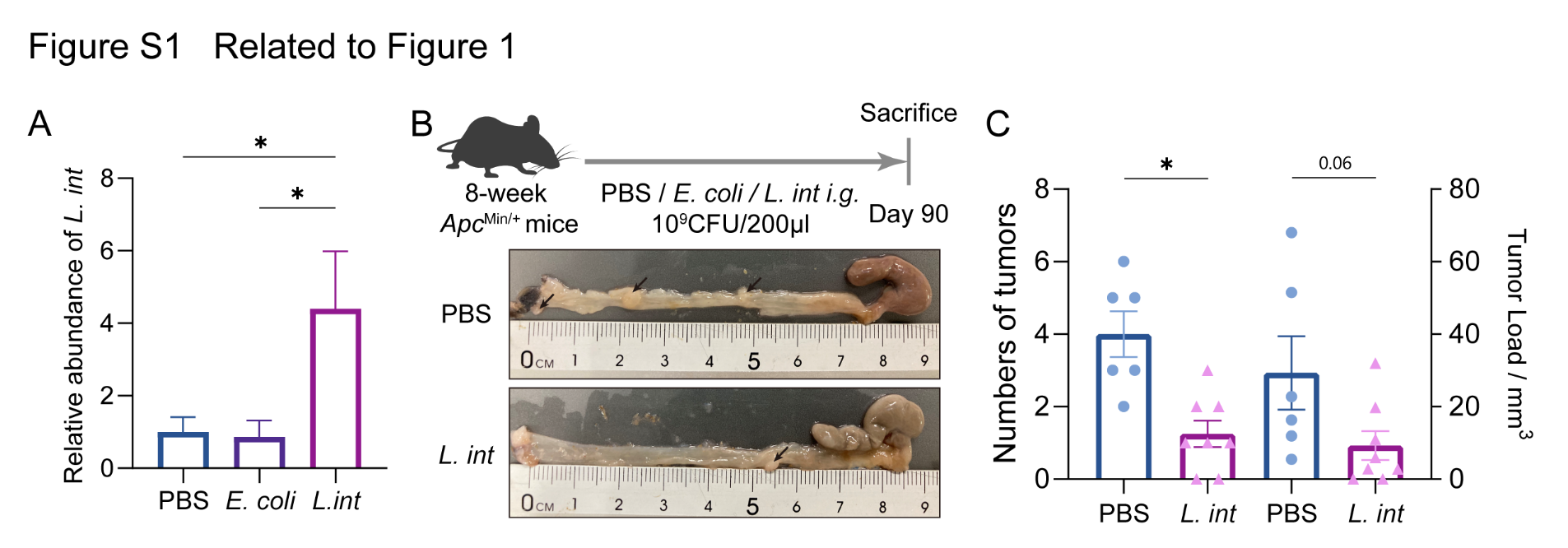


**Figure S1. Supplementation of *L. intestinalis* suppressed colorectal tumorigenesis in mice models**

(A) The abundance of *L. intestinalis* in colon tissue of AOM/DSS-induced CRC mice was measured by bacterial genomic DNA extraction and RT-qPCR (n=9). (B) Schematic diagram showing experimental design for *Apc*^Min/+^ spontaneous adenoma model supplemented with *L. intestinalis,* and representative colorectal images, with arrows showing the tumors. (C) Evidence of tumor suppressive effects of *L. intestinalis*, including numbers of tumors per colon and tumor loads (n=6-8). Data are shown as mean ± SEM. * P < 0.05; ANOVA test (A), Student’s t test (C).


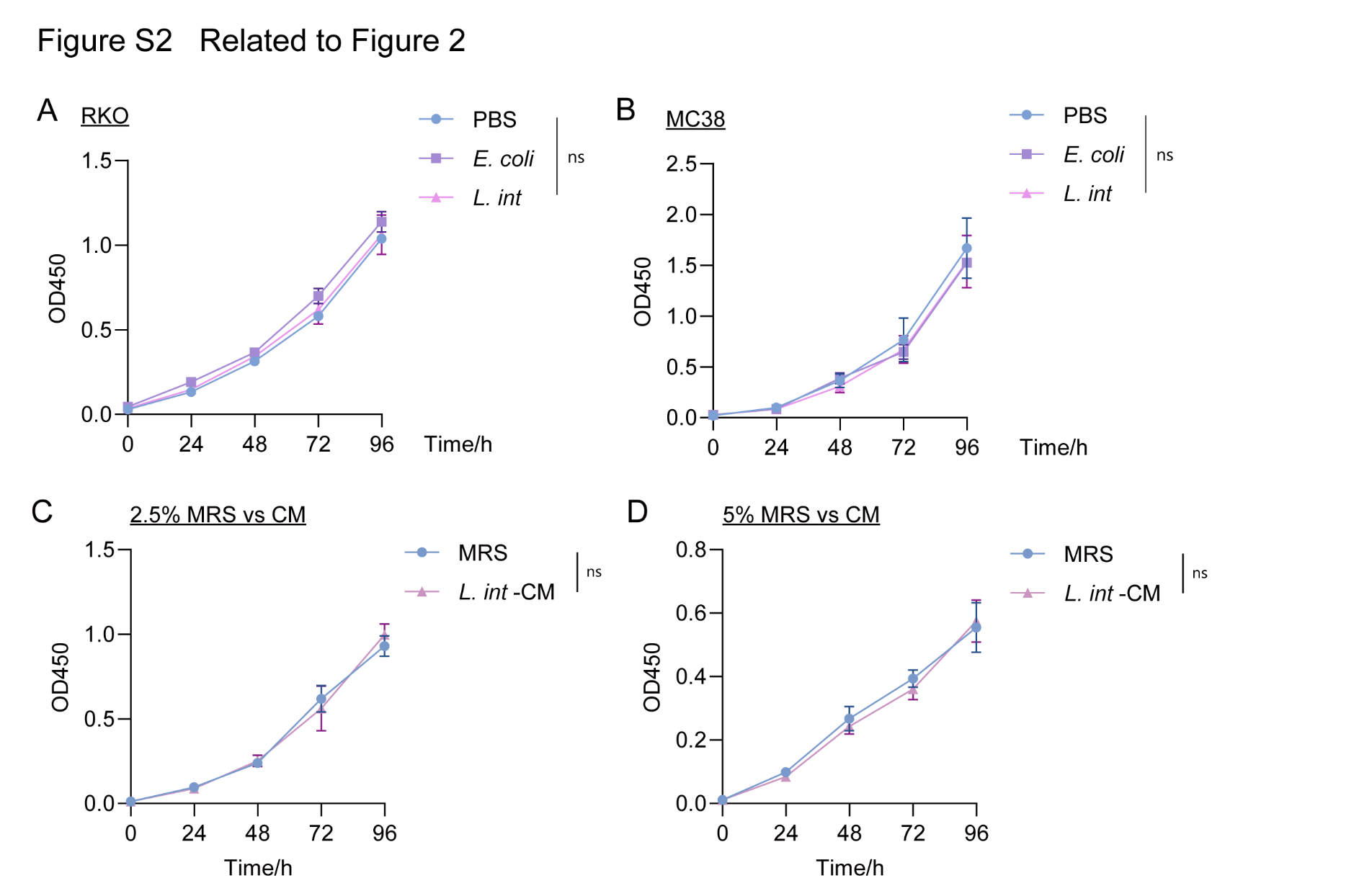


**Figure S2. *L. intestinalis* did not affect tumor cell proliferation *in vitro***

(A) The cell proliferation essay of MC38 cells after co-culturing with PBS, *E. coli,* or *L. intestinalis* for 24 hours (n=3). (B) The cell proliferation assay of RKO cells after co-culturing with PBS, *E. coli*, or *L. intestinalis* for 24 hours (n=3). (C-D) The cell proliferation essay of MC38 cells co-cultured with 2.5%, 5% MRS or conditional medium of *L. intestinalis* (*L. int*-CM). The cell viability was evaluated at 24, 48, 72, and 96 hours with CCK-8 kit (n=3). Data are shown as mean ± SEM. ns, no significance; ANOVA test (A-B), Student’s t-test (C-D).


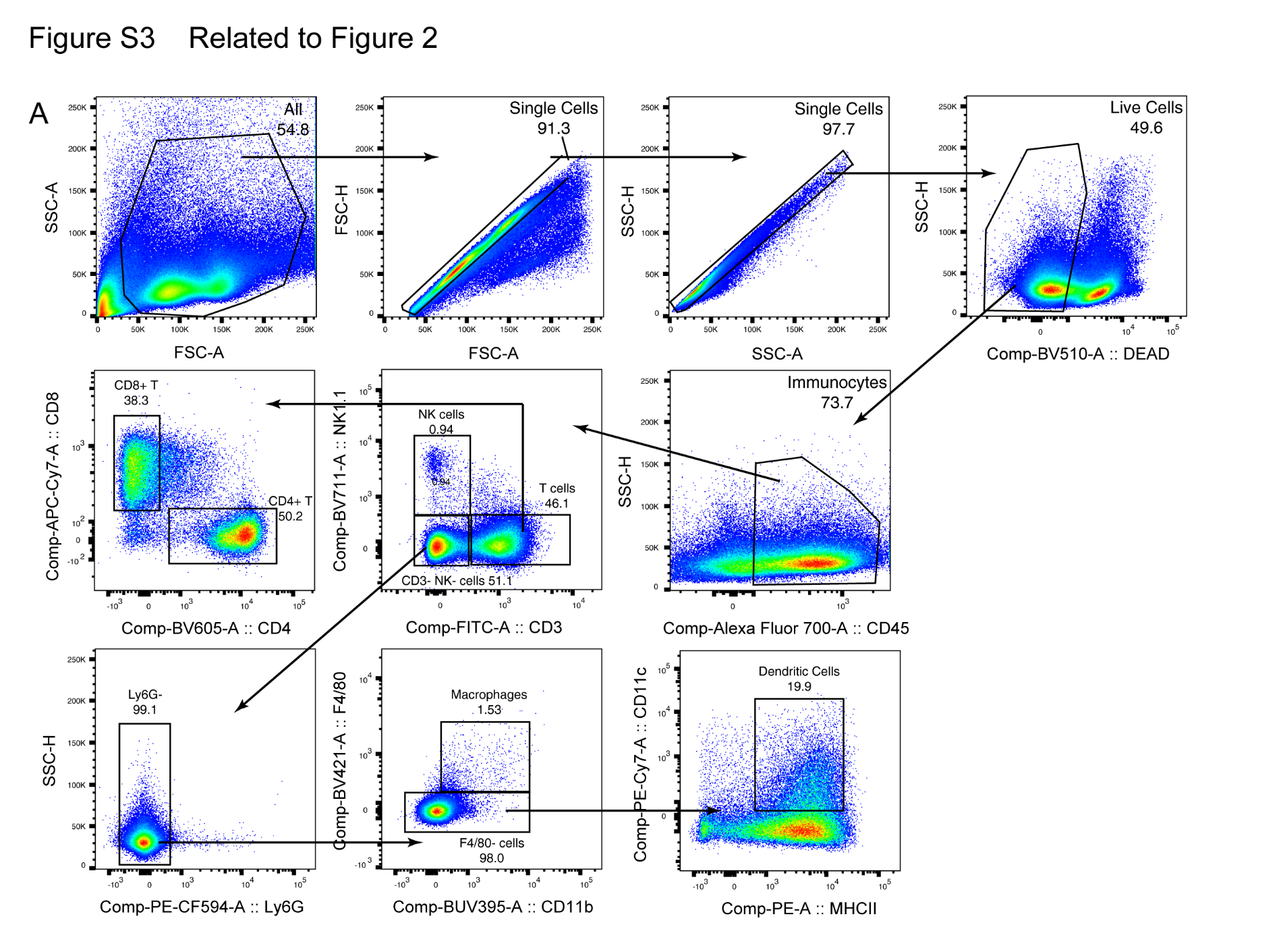


**Figure S3. The gating strategy of flow cytometry for tumor-infiltrated immune cells**

(A) Fixable viability stain 510 was utilized to identify live cells and thereafter CD45 was used for gating total immune cells. Then cells were divided by CD3 and NK1.1 into T cells, natural killer T (NK-T) cells, natural killer (NK) cells, and CD3^-^NK1.1^-^cells. T cells were following identified as CD4^+^ T cells and CD8^+^ T cells. Ly6G was used to exclude granulocytes in CD3^-^NK1.1^-^cells and F4/80^+^ CD11b^+^ cell cluster would be defined as macrophages. Then, dendritic cells (DC) were identified with MHCII^+^ CD11c^+^ in F4/80^-^ cluster.


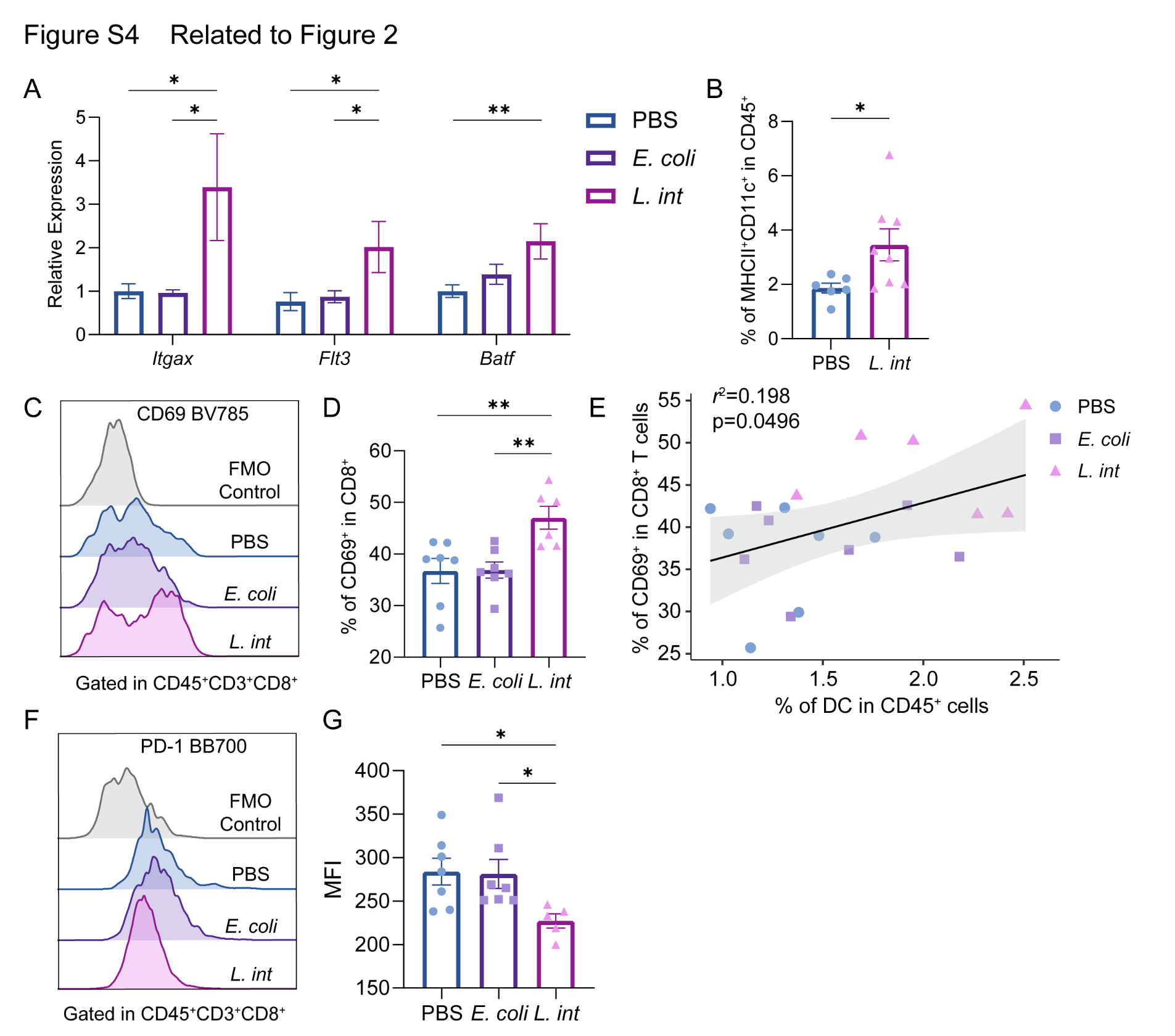


**Figure S4.** ***L. intestinalis* remodeled tumor immune microenvironment in mouse CRC models**

(A) Expression of dendritic cell-related genes *Itgax*, *Flt3*, and *Batf* at mRNA level tested by RT-qPCR, n=9. (B) Percentage of dendritic cells in tumors of *Apc*^Min/+^ spontaneous adenoma model. (C-D) Representative histogram and percentage of CD69^+^ cells in CD8^+^ T cells in tumor of AOM/DSS-induced CRC model (n=6-7). (E) The correlation of the percentage of CD69^+^ cells in CD8^+^ T cells and the percentage of dendritic cells in immune cells (n=6-7). (F-G) Representative histogram of PD-1 expression in CD8^+^ T cells and its mean fluorescence intensity (MFI) in AOM/DSS-induced CRC model (n=6-7). Data are shown as mean ± SEM. *, P < 0.05; **, P < 0.01; ANOVA test (A, D, G), Student’s t test (B), linear regression (E). FMO control, fluorescence-minus-one control.


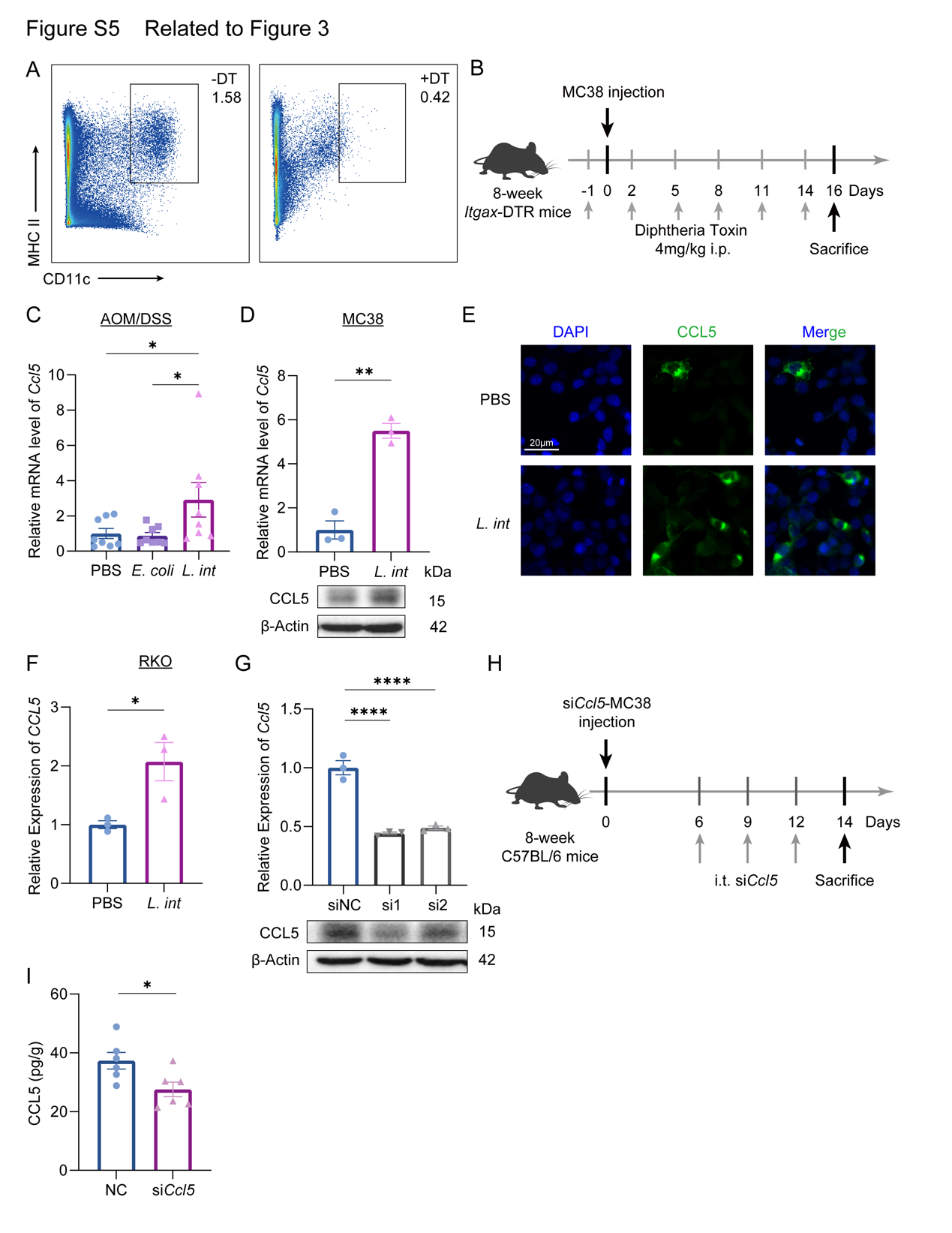


**Figure S5.** **Tumor secreted CCL5 was crucial in *L. intestinalis*-induced DC chemotaxis**

(A) The percentage of dendritic cells (DCs) in *Itgax*-DTR mice after injection with or without DT, as measured by flow cytometry. (B) Schematic representation of the DC-deficient model. MC38 cells, co-cultured with either PBS or *L. intestinalis* for 24 hours, were subcutaneously implanted into *Itgax*-DTR mice following DT administration, with subsequent treatments every 3 days. (C) The mRNA expression levels of *Ccl5* in tumors from AOM/DSS model were tested by RT-qPCR (n=8). (D-E) The expression levels of CCL5 in MC38 cells co-cultured with PBS or *L. intestinalis* was examined by RT-qPCR, western blotting, or immunocytochemistry staining. (F) The expression level of the *CCL5* gene on the mRNA level of RKO cells co-cultured with PBS or *L. intestinalis* was tested by RT-qPCR. (G) Validation of knockdown effect of si*Ccl5* on MC38 cells by RT-qPCR and western blotting. (H) Schematic diagram of *in vivo* si*Ccl5* model. Mice were subcutaneously implanted with MC38 cells transfected with si*Ccl5* or non-target siRNA, and intratumorally injected with si*Ccl5* every 3 days until sacrifice. (I) The concentration of CCL5 in tumor of *in vivo* si*Ccl5* model was measured by ELISA (n=6). *, P < 0.05; **, P < 0.01; ****, P < 0.0001; ANOVA test (C, G), Student’s t test (D, F, H). DT, diphtheria toxin; i.p., intraperitoneal injection. i.t., intratumorally injection.


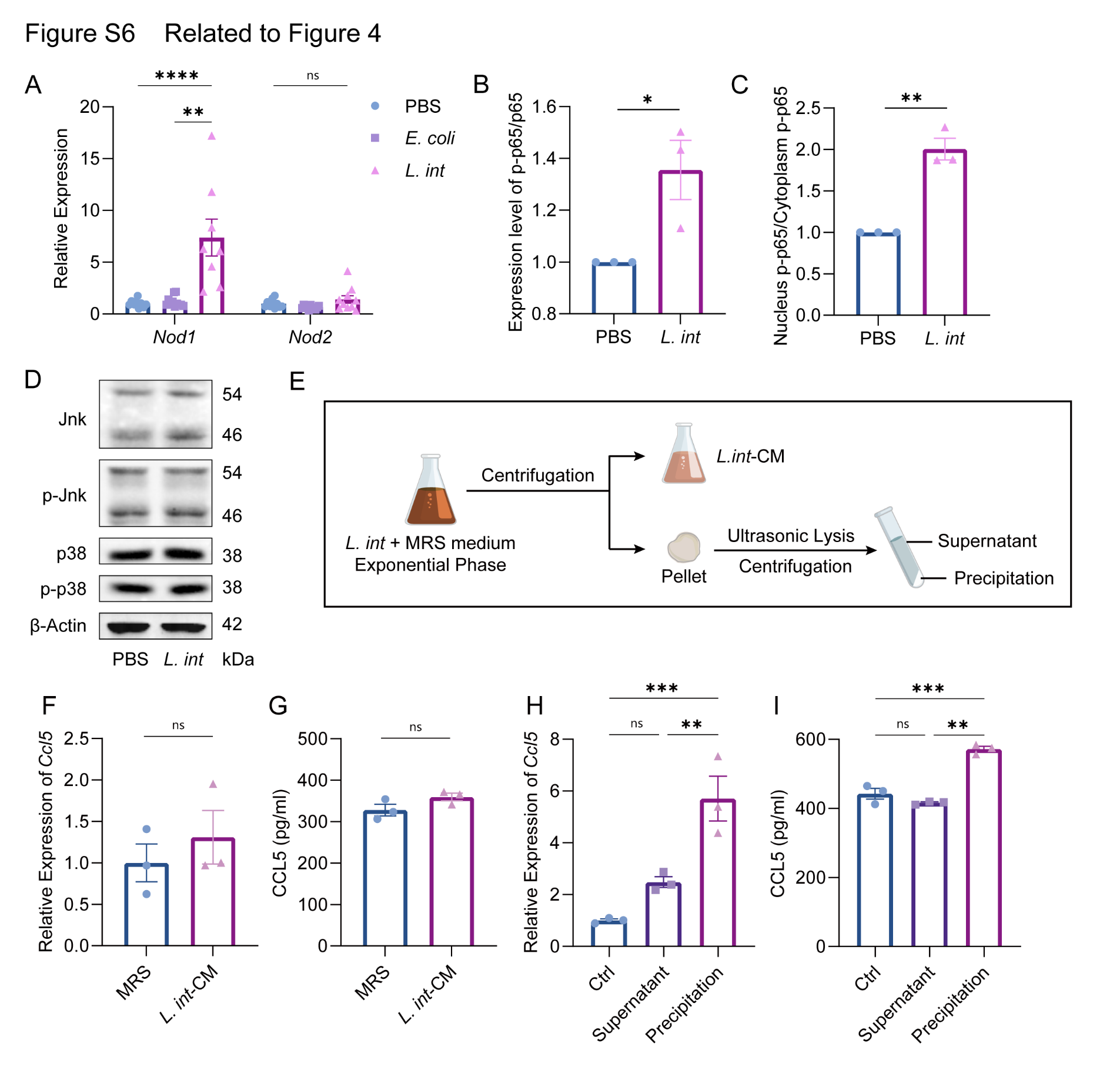


**Figure S6.** **NOD1-NF-κB pathway was involved in *L. intestinalis*-induced CCL5 secretion**

(A) The expression level of *Nod1* and *Nod2* of colorectal tumor in AOM/DSS-induced CRC model tested by RT-qPCR. (B) The expression ratio of phospho-p65 to p65 in MC38 cells co-cultured with PBS or *L. intestinalis* for 24 hours. (C) The expression ratio of nucleus phospho-p65 to cytoplasmic phospho-p65 in MC38 cells co-cultured with PBS or *L. intestinalis* for 24 hours. (D) The expression level of Jnk, phospho-Jnk, p38, and phospho-p38 in MC38 cells co-cultured with PBS or *L. intestinalis* for 24 hours was tested by western blotting. (E) Schematic diagram of isolate conditional medium and ultrasonic lysis fractions of *L. intestinalis*. (F-G) The expression of CCL5 in MC38 co-cultured with MRS or *L. int*-CM for 24 hours quantified by RT-qPCR (F) and ELISA (G). (H-I) The expression of CCL5 in MC38 co-cultured with supernatant or precipitation of ultrasonic lysis of *L. intestinalis* for 24 hours quantified by RT-qPCR (H) and ELISA (I). Data are presented as mean ± SEM, ** P < 0.01; ***, P < 0.001; ****, P < 0.0001; Student’s t-test (B-C, F-G), ANOVA test (A, H-I).

**Table S1. Sequence of siRNA used in *Ccl5* knockdown**

| Name | Sequence (5’-3’) |
| --- | --- |
| si*Ccl5*-sense | CAGAGAAGAAGUGGGUUCATT |
| si*Ccl5*-antisense | UGAACCCACUUCUUCUCUGTT |
| Negative Control-sense | UUCUCCGAACGUGUCACGUTT |
| Negative Control-antisense | ACGUGACACGUUCGGAGAATT |

**Table S2. Primers used to assess gene expression levels and quantify bacterial abundance**

| Gene | Sequence (5’-3’) |
| --- | --- |
| *L. intestinalis*-F | ATCCGCTAGAAGCTGTGGAAA |
| *L. intestinalis* -R | AATCACCTGCATACACGGCT |
| *universal Eubacteria 16s*-F | CGGCAACGAGCGCAACCC |
| *universal Eubacteria 16s*-R | CCATTGTAGCACGTGTGTAGCC |
| Mouse *Actb*-F | GAGACCTTCAACACCCCAGC |
| Mouse *Actb*-R | GGAGAGCATAGCCCTCGTAGAT |
| Mouse *Itgax*-F | GATTGATGGCTCGGGTAGCA |
| Mouse *Itgax* -R | CTGCATCAGGGAGAACCGTG |
| Mouse *Flt3*-F | GCTGTACGTGCTAAGAAGACC |
| Mouse *Flt3*-R | AGCATCTGATGTCTGTTCCGA |
| Mouse *Batf*-F | CCCTGGCAAACAGGACTCATC |
| Mouse *Batf* -R | GGTGTCGGCTTTCTGTGTCT |
| Mouse *Ccl5*-F | CTCCAATCTTGCAGTCGTGTTT |
| Mouse *Ccl5* -R | AGAGCAAGCAATGACAGGGAA |
| Mouse *Nod1*-F | AGCTGGAGGATGCTTACGTG |
| Mouse *Nod1* -R | AGCTGGAGGATGCTTACGTG |
| Mouse *Nod2*-F | AAGCCCTAGCACTGATGCTG |
| Mouse *Nod2* -R | TGCCATTGTTGGACAGTTTCAA |
| Human *ACTB*-F | AGAGCTACGAGCTGCCTGAC |
| Human *ACTB*-R | AGCACTGTGTTGGCGTACAG |
| Human *CCL5*-F | CCTGCTGCTTTGCCTACATTGC |
| Human *CCL5*-R | ACACACTTGGCGGTTCTTTCGG |
| Human *ITGAX*-F | TCCTTCGAATTGGAGATGGCA |
| Human *ITGAX* -R | TCGGTGGAGTAACCCAGGTA |
| Human *NOD1*-F | GGAGGTGTCCGAGTTCTTCC |
| Human *NOD1*-R | GGGTATACCTGCTCACTGGG |
